# Supplementary material for: Peptidomic analysis of CSF reveals new biomarker candidates for amyotrophic lateral sclerosis
Source: EMBO Mol Med. 2025 Jul 18;17(8):1926–49. doi: 10.1038/s44321-025-00272-w (PMC12340150; doi:10.1038/s44321-025-00272-w)
Supplement: Supplementary file 2 — Appendix [file 44321_2025_272_MOESM2_ESM.pdf]

Appendix

Peptidomic analysis of CSF reveals new biomarker candidates for amyotrophic lateral sclerosis

Besnik Muqaku[1], Johannes Dorst[1,2], Maximilian Wiesenfarth[2], Markus Otto[3], Albert C. Ludolph[1,2] and, Patrick Oeckl[1,2]

1 German Center for Neurodegenerative Diseases (DZNE e.V.), 89081 Ulm, Germany
2 Department of Neurology, Ulm University Hospital, 89081 Ulm, Germany
3 Department of Neurology, Martin-Luther-University Halle-Wittenberg, 06120 Halle (Saale), Germany

Appendix Table	Description	Page
S1	Number and percentage of tryptic peptides in Con and ALS, as well as p-value for the comparison between conditions.	2
S2	Fold change and p-value for label-free PRM data from the discovery cohort.	3
S3	Fold change and p-value for PRM data from the validation cohort.	4
S4	P-value for the correlation of eight peptides and clinical data in all patients from the validation cohort.	5
S5	Fold change and p-value for PRM data from the neurodegeneration cohort.	6

Appendix Table S1. Number and percentage of tryptic peptides in Con and ALS, as well as p-value for the comparison between conditions.

Tryptic peptides	Number (Average)	% (Average)
Con	234.83	2.08%
ALS	268.67	2.37%
p-value	0.006905	0.00823

Appendix Table S2. Fold change and p value for label-free PRM data from the discovery cohort. Fold change > 1 indicates an increase in ALS.

Label-free PRM	NFL	NFM_VQ	NFM_LK	MAP1B	MYL1	APOC1_SE	APOC1_TP	CO3	PENK	CADM3	SCG1
Fold change (AverageALS/AverageCon)	18.35026516	14.29366682	23.04022	2.55824	7.118307	2.700634	3.627777	2.46374	0.529556	0.710211	0.674375
p-value	0.000003093	0.000003093	3.09E-06	0.000832	0.001129	0.008957	0.003436	0.01115	0.02058	0.02492	0.02492

Appendix Table S3. Fold change and p value for PRM data from the validation cohort. Log2 fold change > 0 indicates an increase in A for the comparison A vs. B.

Fold change (Average A (log2) - Average B (log2))	NFL	MAP1B_KE	MAP1B_EA	MYL1	APOC1	CADM3	SCG1	PENK	Figure
ALS-Con	3.794251734	1.002581429	0.92606682	2.579741121	1.225516	-0.330692143	-0.409599	-0.141923	4A
sALS-Con	3.703223766	0.955230784	0.877867593	2.315102597	1.22186	-0.336369232	-0.359326	-0.074663	4B, 4C
gALS-Con	3.968392194	1.091106548	1.018274037	3.086006122	1.23251	-0.319831625	-0.505773	-0.270594	4B
C9orf72-Con	4.569467885	1.270030809	1.018355902	2.509740329	0.983624	-0.503244936	-0.644495	-0.501318	4C
SOD1-Con	3.312673258	0.895916446	1.018184729	3.714659715	1.504022	-0.119744376	-0.354439	-0.018895	4C
C9orf72-sALS	0.86624412	0.314800025	0.140488309	0.194637731	-0.238236	-0.166875704	-0.285169	-0.426654	4C

p-value	NFL	MAP1B_KE	MAP1B_EA	MYL1	APOC1	CADM3	SCG1	PENK	Figure
ALS-Con	1.25E-14	3.20E-09	2.59E-09	5.28E-08	7.84E-09	0.0005951	0.002609	0.04912	4A
sALS-Con	2.60E-11	1.50E-06	9.10E-07	3.60E-05	2.00E-07	0.0068	0.0483	0.7606	4B
gALS-Con	1.80E-09	1.00E-06	1.50E-06	4.30E-07	9.00E-05	0.0186	0.0139	0.0334	4B
sALS-Con	5.20E-11	2.90E-06	1.80E-06	7.30E-05	4.00E-07	0.0136	0.0966	1	4C
C9orf72-Con	1.20E-07	1.30E-04	0.00022	0.0028	0.0289	0.0209	0.0426	0.0035	4C
SOD1-Con	0.00037	0.00133	0.00132	7.50E-06	0.0012	1	0.5391	1	4C
C9orf72-sALS	1	1	1	1	1	1	1	0.0416	4C

Appendix Table S4. P-value for the correlation of eight peptides and clinical data in all patients from the validation cohort.

NFL										
MAP1B_KE	2.2E-16									
MAP1B_EA	2.2E-16	2.2E-16								
MYL1	2.8E-08	1.4E-07	2.6E-08							
APOC1	1.0E-08	1.6E-07	1.6E-06	3.0E-07						
CADM3	0.011	0.38	0.12	0.044	0.0025					
SCG1	0.021	0.37	0.49	0.0068	0.06	3.2E-16				
PENK	0.07	0.8	0.54	0.12	0.27	3.7E-09	2.6E-12			
Age	0.0043	0.11	1.5E-04	0.46	0.51	0.39	0.15	0.21		
FRS_r	0.0015	9.8E-04	0.015	0.82	0.24	0.78	0.55	0.91	0.8	
D.duration	0.049	0.13	0.54	0.77	0.96	0.47	0.69	0.88	0.53	0.94
	NFL	MAP1B_KE	MAP1B_EA	MYL1	APOC1	CADM3	SCG1	PENK	Age	FRS_r	D.duration

Appendix Table S5. Fold change and p value for PRM data from the neurodegeneration cohort. Log2 fold change > 0 indicate increase in A when comparing A vs. B.

Fold change (Average A (log2) - Average B (	NFL	MAP1B_EA	MYL1	APOC1	CADM3	SCG1	PENK
AD-Con	0.7057989	0.287993	0.290282	0.6184808	-0.20768	-0.228919	0.0796847
bvFTD-Con	1.4708823	0.3613312	1.1219351	0.4418012	0.053402	-0.086692	-0.168508
PD-Con	-0.204558	0.3001565	0.4618103	0.2159501	-0.075692	0.1338666	-0.146093
AD-PD	0.9103571	-0.012163	-0.171528	0.4025307	-0.131987	-0.362786	0.2257776
bvFTD-PD	1.6754404	0.0611748	0.6601248	0.2258511	0.1290943	-0.220559	-0.022415

p-value	NFL	MAP1B_EA	MYL1	APOC1	CADM3	SCG1	PENK
AD-Con	0.26913	1	1	1	1	1	1
bvFTD-Con	0.00931	1	0.4673	1	1	1	1
PD-Con	1	1	1	1	1	1	1
AD-PD	0.03351	1	1	1	1	0.611	1
bvFTD-PD	0.00068	1	1	1	1	1	1
