# Supplementary material for: Peptidomic analysis of CSF reveals new biomarker candidates for amyotrophic lateral sclerosis
Source: EMBO Mol Med. 2025 Jul 18;17(8):1926–49. doi: 10.1038/s44321-025-00272-w (PMC12340150; doi:10.1038/s44321-025-00272-w)
Supplement: Supplementary file 10 — Expanded View Figures [file 44321_2025_272_MOESM10_ESM.pdf]

# Expanded View Figures

**Figure EV1. Characterization of the MYL1 peptide.**

Targeted PRM chromatograms of a CSF pool sample spiked with the MYL1 stable isotope-labeled standard peptides (SISPep). Acetylated MYL1 SISPeps (blue, top and in the middle) eluted almost 1.5 min later than the endogenous MYL1 peptide (red). The trimethylated MYL1 SISPep (blue, bottom) eluted exactly at the same time as endogenous MYL1 peptide (red) and confirmed the identity of MYL1 peptide.

 © The Author(s)

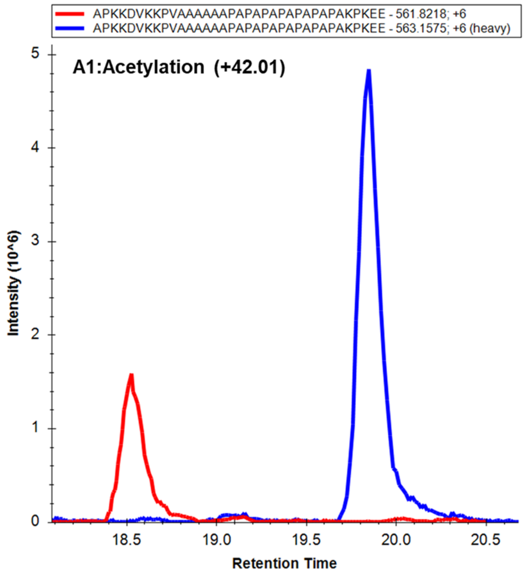
APKKDVKKPVAAAAAAPAPAPAPAPAPAPAKPKEE - 561.8218; +6
APKKDVKKPVAAAAAAPAPAPAPAPAPAPAKPKEE - 563.1575; +6 (heavy)
A1:Acetylation (+42.01)
Intensity (10^6)
0
1
2
3
4
5
18.5
19.0
19.5
20.0
20.5
Retention Time

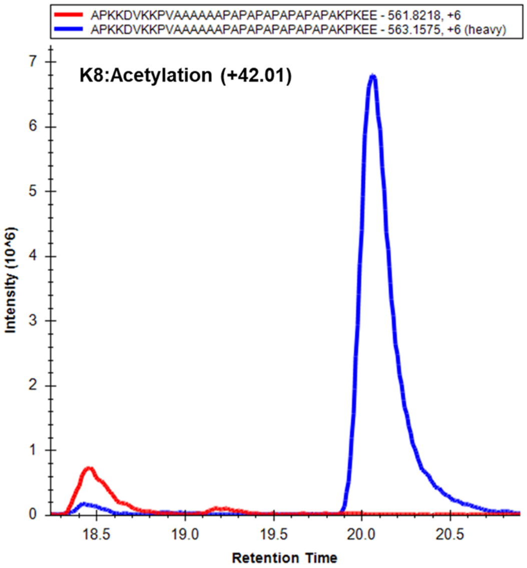
APKKDVKKPVAAAAAAPAPAPAPAPAPAPAKPKEE - 561.8218, +6
APKKDVKKPVAAAAAAPAPAPAPAPAPAPAKPKEE - 563.1575, +6 (heavy)
K8:Acetylation (+42.01)
Intensity (10^6)
0
1
2
3
4
5
6
7
18.5
19.0
19.5
20.0
20.5
Retention Time

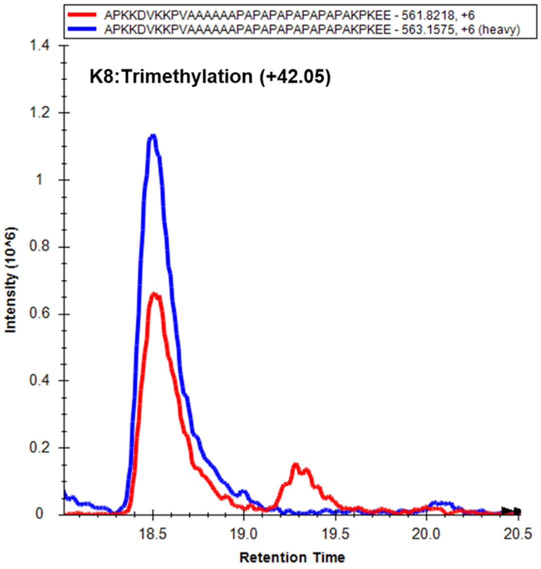
APKKDVKKPVAAAAAAPAPAPAPAPAPAPAKPKEE - 561.8218, +6
APKKDVKKPVAAAAAAPAPAPAPAPAPAPAKPKEE - 563.1575, +6 (heavy)
K8:Trimethylation (+42.05)
Intensity (10^6)
0
0.2
0.4
0.6
0.8
1
1.2
1.4
18.5
19.0
19.5
20.0
20.5
Retention Time

© The Author(s)

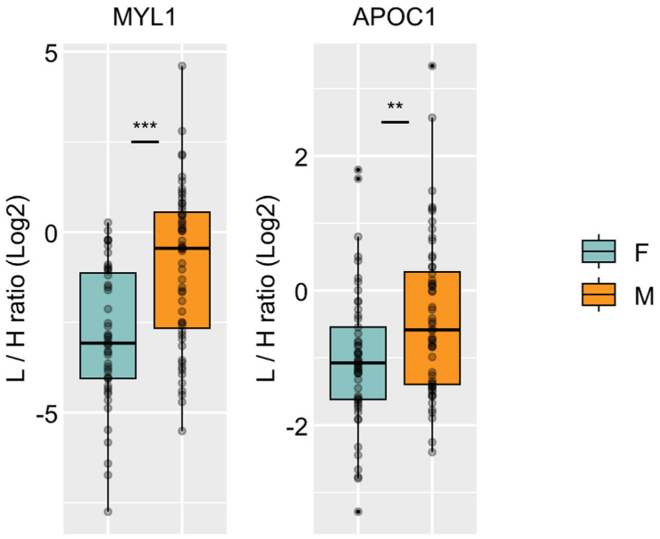


**Figure EV2. Levels of MYL1 and APOC1 in females and males.**

Boxplots show the PRM data for MYL1 and APOC1 by comparing females ($n = 53$) and males ($n = 56$) over all patients from validation cohort. For both peptides the data were normalized to their respective stable isotope-labeled standard peptide (SISPep) indicated as light to heavy ration (L/H ratio). Wilcoxon test was applied for group comparison analysis. Data are visualized by boxplots (minima, maxima, center, bounds of box and whiskers, and percentile) and individual values. Peptide (log2 fold change; *P* value): MYL1(1.97; $1.3*10^{-5}$) and APOC1(0.60; 0.0097). Fold change (average M (log2)-average F (log2)). Log2 fold change >0 indicates an increase in males. **$P$ value < 0.01; ***$P$ value < 0.001.

 © The Author(s)

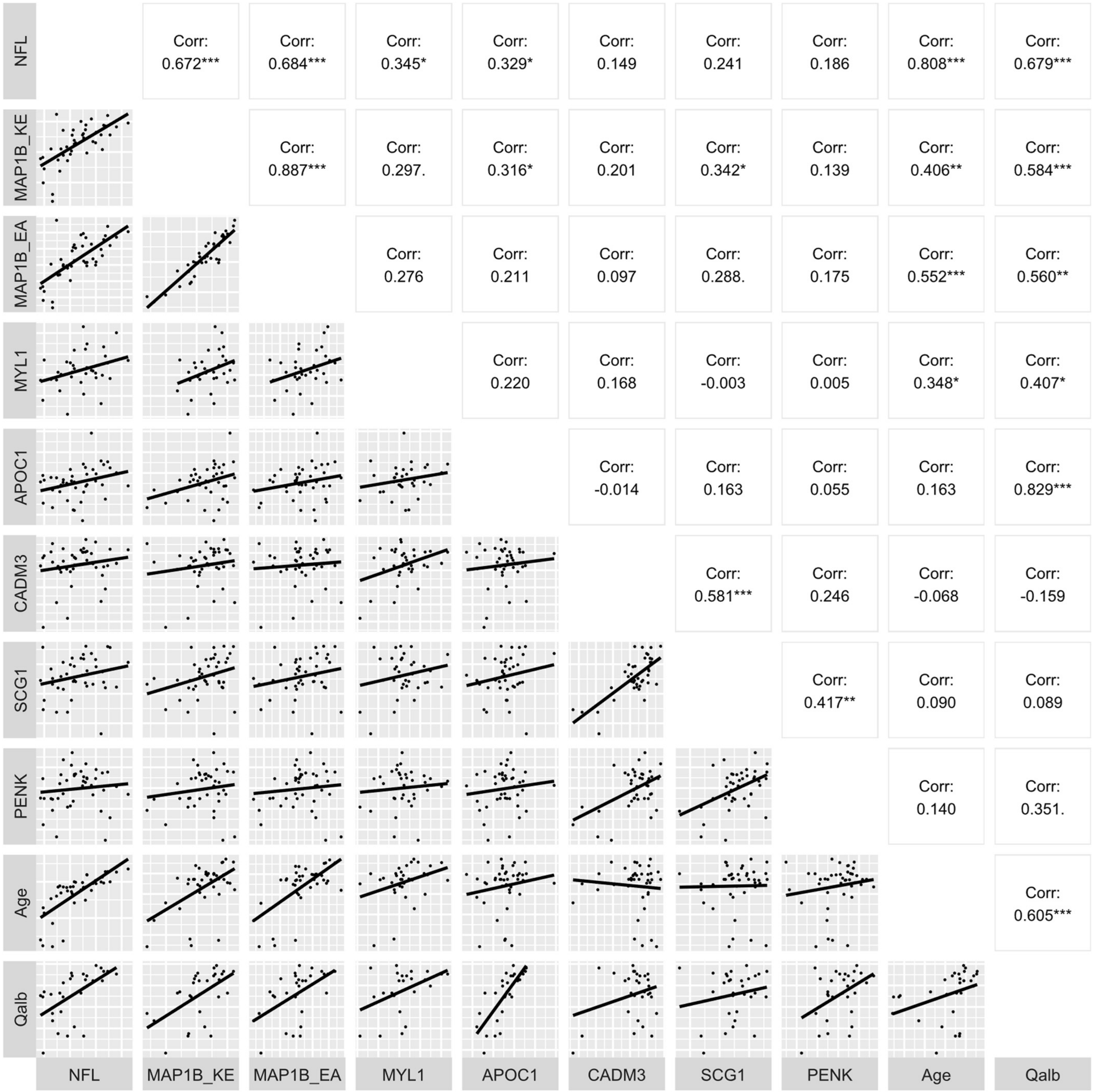


**Figure EV3. Correlation of peptides with clinical data in controls.**

Multi scatterplot show the correlation and Spearman's correlation coefficient for eight peptides and clinical data only in controls from the validation cohort. All data were log2 transformed and each dot represents the data of a patient. The two different MAP1B peptides are indicated by the first two amino acids of the peptide sequence. Qalb - albumin quotient. **P* value < 0.05; ***P* value < 0.01; ****P* value < 0.001.

© The Author(s)

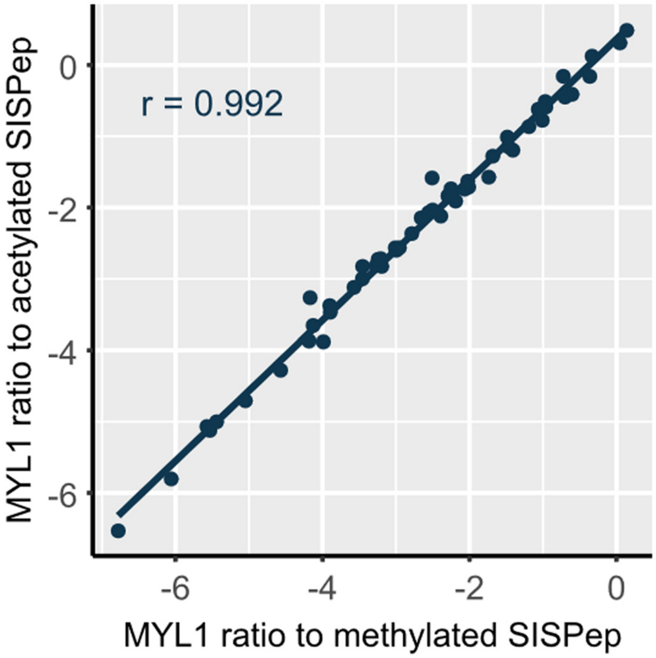


**Figure EV4. Correlation of MYL1 normalized to acetylated and methylated standard.**

Scatterplot show the correlation and Spearman's correlation coefficient for the ratio of MYL1 peptide normalized to acetylated and methylated stable isotope-labeled standard peptide (SISPep) in samples from neurodegeneration cohort. Log2 data.

 © The Author(s)
